# Independent association between meteorological factors, PM2.5, and seasonal influenza activity in Hangzhou, Zhejiang province, China

**DOI:** 10.1111/irv.12829

**Published:** 2020-12-20

**Authors:** Steven Yuk‐Fai Lau, Wei Cheng, Zhao Yu, Kirran N. Mohammad, Maggie Haitian Wang, Benny Chung‐Ying Zee, Xi Li, Ka Chun Chong, Enfu Chen

**Affiliations:** ^1^ School of Public Health and Primary Care The Chinese University of Hong Kong Hong Kong China; ^2^ Zhejiang Province Centre for Disease Control and Prevention Hangzhou China; ^3^ Clinical Trials and Biostatistics Laboratory Shenzhen Research Institute The Chinese University of Hong Kong Hong Kong China; ^4^ Centre for Health Systems and Policy Research The Chinese University of Hong Kong Hong Kong China

**Keywords:** humidity, influenza, PM2.5, rainfall, subtropic, temperature

## Abstract

**Background:**

Due to variations in climatic conditions, the effects of meteorological factors and PM_2.5_ on influenza activity, particularly in subtropical regions, vary in existing literature. In this study, we examined the relationship between influenza activity, meteorological parameters, and PM_2.5_.

**Methods:**

A total of 20 165 laboratory‐confirmed influenza cases in Hangzhou, Zhejiang province, were documented in our dataset and aggregated into weekly counts for downstream analysis. We employed a combination of the quasi‐Poisson‐generalized additive model and the distributed lag non‐linear model to examine the relationship of interest, controlling for long‐term trends, seasonal trends, and holidays.

**Results:**

A hockey‐stick association was found between absolute humidity and the risk of influenza infections. The overall cumulative adjusted relative risk (ARR) was statistically significant when weekly mean absolute humidity was low (<10 µg/m^3^) and high (>17.5 µg/m^3^). A slightly higher ARR was observed when weekly mean temperature reached over 30.5°C. A statistically significantly higher ARR was observed when weekly mean relative humidity dropped below 67%. ARR increased statistically significantly with increasing rainfall. For PM_2.5_, the ARR was marginally statistically insignificant. In brief, high temperature, wet and dry conditions, and heavy rainfall were the major risk factors associated with a higher risk of influenza infections.

**Conclusions:**

The present study contributes additional knowledge to the understanding of the effects of various environmental factors on influenza activities. Our findings shall be useful and important for the development of influenza surveillance and early warning systems.

## 1 INTRODUCTION

Seasonal influenza is a common respiratory tract viral infection that might lead to localized outbreaks or even pandemic, having substantial impacts on human health as well as societies and economies. According to the World Health Organization (WHO), an estimated 290 000‐650 000 deaths were associated with influenza globally in 2017.^1^, ^2^ In the United States, the estimated mean annual total cost of influenza reached $11.2 billion.^3^ Seasonal influenza imposed a significant economic burden to the healthcare system and society. While environmental factors are typically regarded as strong drivers of influenza epidemic, inconsistency in findings was evident in existing literature possibly due to variations in climatic conditions. Thus, there is a need to provide additional insights relevant to influenza seasonality. A reliable epidemic forecast is crucial to optimize vaccination strategy, strengthen healthcare resource preparedness, and ultimately ease the disease burden of influenza.

Several studies have assessed the effects of weather on influenza activity. Tamerius et al^4^ explored the association between climate variability and timing of seasonal influenza epidemics in 78 study sites sampled globally. Results suggested that “cold‐dry” and “humid‐rainy” conditions tend to be associated with influenza seasonality worldwide.^4^ Using guinea pig as the model host, Lowen et al^5^ showed that low temperature and low humidity favor influenza transmission. In another study conducted in Shanghai, Hong Kong, and British Columbia, influenza peaks were observed at temperature extremes as well as high relative humidity.^6^ While relative humidity is a metric of water vapor relative to ambient temperature, absolute humidity is independent of ambient temperature. Some studies conducted in temperate settings indicated that absolute humidity alone could already modulate influenza seasonality at population level.^7^, ^8^ Similar studies also supported a strong relationship between absolute humidity and influenza activity in temperate cities,^9^, ^10^ although it is argued that relative humidity may be more likely to modulate the survival of the virus in aerosols.^11^ In tropical and subtropical settings where temperature and relative humidity are generally higher than that in temperate settings, their impacts on influenza activity may not be consistent. Previous studies showed influenza activities in Hong Kong, Brazil, and India were higher during rainy seasons,^12^, ^13^, ^14^ while influenza activity in Singapore was not statistically significantly related to rainfall.^15^

Air pollutants such as ambient fine particulate matter (PM_2.5_) have been shown to be environmental triggers of seasonal influenza epidemics.^16^, ^17^, ^18^, ^19^ In China, over 1.3 billion people are at high health risks due to exposure to PM_2.5_ in concentrations exceeding the WHO Air Quality Guidelines.^20^ However, the findings were rather inconsistent across studies. A study conducted by Ma et al^21^ using in vivo PM_2.5_ mouse pharyngeal wall drop‐in model demonstrated that long‐term exposure to PM_2.5_ would lower one's ability to combat the influenza virus through down‐regulating pulmonary macrophage Kdm6a and mediating histones modification in IL‐6 and IFN‐β promoter regions. Two epidemiological studies conducted by Feng et al^17^ and Liang et al^19^ utilized the wavelet approach and generalized additive models to explore the relationship between PM_2.5_ and influenza in Beijing, China, and revealed a significant positive association between the two. These findings were further supported by another multi‐city study in China where PM_2.5_ was reported to be associated with an increased risk of influenza exposure especially on cooler days.^22^ However, a population‐based study in Nanjing, China, found a null or negative effect of ambient PM_2.5_ on daily influenza cases.^23^

The diversified results in current literature might be due to variations in climatic conditions. Given environmental effects documented in subtropical settings are fairly inconsistent, we employed a multivariable model to elucidate the impacts of different meteorological factors and PM_2.5_ on influenza activity in a subtropical city in China with non‐linear and lagged effects taken into account. Our findings could provide additional insights relevant to influenza seasonality which would, ultimately, help improve prevention and control strategies.

## 2 METHODS

### 2.1 Study site and Influenza data

Hangzhou (29°11′‐30°34′N and 118°20′‐120°37′E) is a subprovincial city of Zhejiang Province, an eastern coastal province of China with humid subtropical climate and four distinct seasons. Hangzhou covers a total area of 16 595 km^2^, subdivided into 10 districts, two counties, and one city, with a permanent resident population of 9.47 million at the end of 2017. Surveillance on seasonal influenza viruses was performed by a web‐based notifiable infectious disease management system (China Information System for Disease Control and Prevention (CISDCP)).^24^ A total of 39 notifiable infectious diseases were monitored by this surveillance system, and they were divided into three categories—classes A, B, and C—all of which must be reported within a specified timeframe. All class A infectious diseases and some class B diseases (e.g. pulmonary anthrax, severe acute respiratory syndrome, poliomyelitis, and highly pathogenic avian influenza) should be reported to the surveillance system within 2 hours of diagnosis, whereas other class B and class C infectious diseases should be reported within 24 hours. Influenza is a class C notifiable infectious disease in China. The diagnosis of clinically and laboratory‐confirmed influenza infections should meet the criteria issued by the Ministry of Health of the People's Republic of China. All laboratory‐confirmed influenza case records from January 1 2014, to December 31 2018, were anonymously extracted for analysis in this study.

### 2.2 Environmental data

Daily concentrations of ambient fine particulate matter (PM_2.5_ in µg/m^3^) recorded by the 16 monitoring stations in Hangzhou were downloaded from the webpage of China Air Quality Online Monitoring and Analysis Platform (http://www.aqistudy.cn/). Daily meteorological data including daily mean temperature (°C), daily mean relative humidity (%), and daily total rainfall (mm) measured by the three weather stations in Hangzhou were obtained from the China Meteorological Data sharing service system of the China Meteorological Administration (http://data.cma.gov.cn). Daily mean absolute humidity (g/m^3^) was derived from daily mean temperature and daily mean relative humidity using the formulas below.^25^

AH=100×C×Pw/(TEMP+273.15)

Pw=Pws×RH%

$$\text{Pws}=6.112\times \exp\left(\frac{17.67\times TEMP}{TEMP+243.5}\right)$$

AH: absolute humidity (g/m^3^);
C: constant (2.16679 gk/J);
Pw: vapor pressure (hPa);
*TEMP*: temperature (°C);
Pws: saturation vapor pressure (hPa).

For each of the environmental variables, the values measured by different stations on the same day were averaged to obtain a number representing the exposure of a general citizen in the city.

### 2.3 Holiday data

Holiday was found to be associated with seasonal influenza epidemic patterns given its ability to change human contact pattern and was therefore adjusted in the model.^26^ Three kinds of holidays were taken into consideration, including summer and winter vacations as well as Chinese National Day.

### 2.4 Statistical analysis

In this study, all data were aggregated to the weekly level for analysis to smooth the stochastic uncertainty of daily data. Weekly counts of laboratory‐confirmed influenza cases were modeled using a combination of the quasi‐Poisson‐generalized additive model (GAM)^27^ and the distributed lag non‐linear model (DLNM),^28^ with meteorological parameters and PM_2.5_ as covariates while controlling for long‐term and seasonal trends and holidays. The model allowed simultaneous analysis of non‐linear and lagged relationships, both of which are common in time series studies of environmental variables and health outcomes. To further elaborate, weekly counts of laboratory‐confirmed influenza cases were regressed on the environmental exposures (ie, PM_2.5_, temperature, relative humidity (RH), and rainfall) as in the following primary model.

$$\begin{array}{cc} \text{Log}({\text{E[Y}}_{t}])=\beta_{0} & +\text{cb}({\text{sqrt(RF}}_{t}),{\text{df}}_{\text{RF}},\text{lag}=1\text{week},{\text{df}}_{\text{lag}})\\ & \;+{\text{cb(TEMP}}_{t},{\text{df}}_{\text{TEMP}},\text{lag}=1\text{week},{\text{df}}_{\text{lag}})\\ & \;+{\text{cb(RH}}_{t},{\text{df}}_{\text{RH}},\text{lag}=1\text{week},{\text{df}}_{\text{lag}})\\ & \;+\text{cb}(\text{PM}2.5_{t}{\text{,df}}_{\text{PM}2.5},\text{lag}=1\text{week},{\text{df}}_{\text{lag}})\\ & \;+s({\text{WOY}}_{t})+s({\text{Year}}_{t})\\ & \;+{\text{cb(HOLIDAY}}_{t},{\text{df}}_{H},\text{lag}=1\text{weeks},{\text{df}}_{\text{lag}})\\ & \;+\text{autoregressive terms} \end{array}$$

cb(.): cross‐basis function of independent variables built up using the dlnm package in R;
s(.): smoothing spline function of independent variables;
sqrt(RF_t_): square root of total rainfall in week t (1,2,…,260);
TEMP_t_: average ambient temperature in week t;
RH_t_: average relative humidity in week t;
PM2.5_t_: average concentration of PM_2.5_ in week t;
HOLIDAY_t_: number of days of holidays in week t;
df_RF,_ df_TEMP,_ df_RH,_ df_PM2.5_ df_H_ : degrees of freedom of square root of total rainfall, temperature, relative humidity, PM_2.5_, and number of days of holidays of the natural spline function in cb(.) respectively;
df_lag_: degree of freedom of lag of the natural spline function in cb(.);
WOY_t_: week of the year of week t (1,2,…,52 or 53)
Year_t_ : year of week t (2014, 2015,…, 2018)

Since temperature is highly positively correlated with absolute humidity (AH), a secondary model with temperature and RH replaced by AH was fitted in order to assess the independent effect of AH on the response. In both models, total rainfall was square‐rooted to reduce its skewness and impact of outliers. A maximum lag of one week was defined for meteorological and pollutant variables based on average influenza incubation period and laboratory detection time.^29^ Degrees of freedom (dfs) in the models were selected through minimization of generalized cross validation. A maximum df of 7 per year was chosen to account for annual seasonal trend as in similar studies.^30^ Autoregressive terms were added in the model to account for residual autocorrelation. The estimated effects of the environmental factors were reported in the form of overall cumulative adjusted relative risk (ARR) with corresponding 95% confidence interval (CI). For rainfall and PM2.5, the lowest recording or measurement was taken as the reference value, whereas for temperature, RH, and AH, the value at which the minimum risk was observed was taken as the reference value.

### 2.5 Sensitivity analysis and model checking

Sensitivity analyses were performed by altering the maximum lag weeks (2 weeks) to assess the robustness of our results. Goodness‐of‐fit of the models was checked by mean absolute error (MAE) as well as residual analysis. Partial autocorrelation plots were used to examine residual autocorrelation. Statistically insignificant partial autocorrelation function (PACF) for 4 lag weeks was deemed adequate.

All statistical analyses were performed using the *mgcv* and *dlnm* packages in R statistical environment version 3.6.0.^27^

## 3 RESULTS

**Figure 1:**
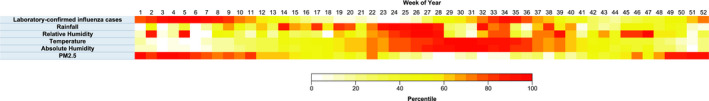
Temporal distributions of weekly total laboratory‐confirmed influenza cases, and average meteorological and pollution parameters from January 1 2014, to December 31 2018, in Hangzhou, Zhejiang, China

Between January 1 2014 and December 31 2018, a total of 20 165 laboratory‐confirmed influenza cases were reported in Hangzhou. The temporal distribution of laboratory‐confirmed influenza cases during the study period was shown in Figure 1. Eight influenza peaks, which appeared every winter to spring and occasionally summer (2014, 2015, and 2017), were observed during the study period. Winter peaks were generally higher than summer peaks. The temporal distributions and summary statistics of weekly meteorological factors and PM_2.5_ were shown in Figure 1 and Table 1, respectively. The seasonal pattern was more observable for temperature, AH, and PM_2.5_ compared with rainfall and RH.

**Table 1:** Summary statistics of weekly air pollutant and meteorological parameters from 2014 to 2018 in Hangzhou, Zhejiang, China

Weekly air pollutant/meteorological parameter	Percentile						
	Min.	5%	25%	50%	75%	95%	Max.
Particulate matter 2.5 (µg/m^3^)	12.29	21.56	33.93	46.36	59.75	91.18	135.57
Temperature (°C)	−0.80	4.60	10.38	18.69	24.49	29.97	32.74
Relative humidity (%)	51.10	61.77	70.60	77.76	83.58	89.01	94.71
Rainfall (mm)	0.00	0.05	6.02	22.33	43.90	108.74	172.83
Absolute humidity (g/m^3^)	3.15	4.61	7.25	12.01	18.48	22.61	23.88

**Figure 2:**
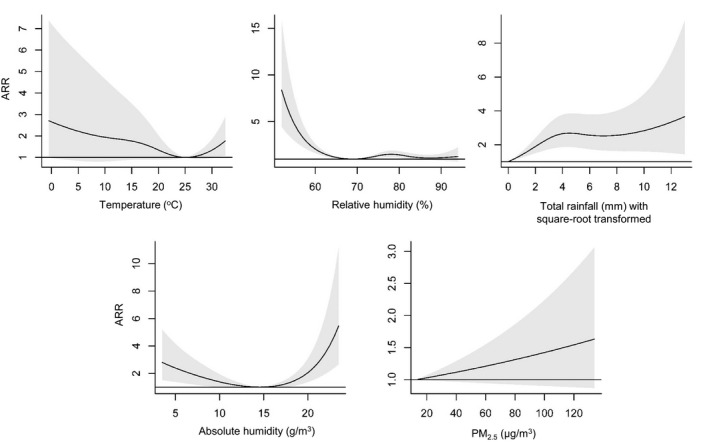
Overall cumulative adjusted relative risk (ARR) and corresponding 95% confidence interval for influenza activity at different levels of pollution and meteorological conditions

The overall cumulative ARRs over a lag of one week against different environmental factors were shown in Figure 2. Based on the primary model, the adjusted risk increased with increasing rainfall, with ARRs being statistically significant over the whole observed range. The ARR was 2.12 (95% CI: 1.61‐2.79) when the weekly total rainfall attained 6.0 mm (25th percentile), while it was 2.53 (95% CI: 1.68‐3.79) when the weekly total rainfall attained 43.9 mm (75th percentile). On the other hand, a hockey‐stick association was found between temperature and influenza activity. Using 25.0°C, at which the lowest adjusted risk was detected, as reference, the adjusted risk was slightly higher at temperature extremes with statistically significant ARRs observed as temperature went above 30.5°C (ARR = 1.44, 95% CI: 1.00‐2.08 at 30.5°C). For RH, the lowest adjusted risk was detected at 69%. When using that point as reference, the ARR was statistically significant as RH dropped below 67%. At the 5th percentile of RH (61.8%), the ARR was   1.63 (95% CI: 1.37‐1.94). Although the ARR was also statistically significantly higher within the RH range of 72%‐82%, the effect size is comparatively small. For PM_2.5_, although the ARR increased with increasing concentration, it was marginally statistically insignificant.

The independent effect of AH was determined based on the secondary model. Apparently, the pattern of AH was similar to that of temperature and RH: the adjusted risk was relatively higher at humidity extremes. Further examination showed that the lowest risk was detected at 14.5 µg/m^3^, and the adjusted risk was statistically significant when AH was low (<10 µg/m^3^) and high (>17.5 µg/m^3^) when compared to that point.

Sensitivity analysis, which was performed by changing the maximum lag of all environmental variables to 2 weeks, gave results that were consistent with those in our main analysis and were shown in Figure S1. For model diagnosis, MAE of the primary model was 11.19. Residual analysis showed that all models have statistically insignificant PACFs for 4 lag weeks, indicating an acceptable level of residual autocorrelation.

## 4 DISCUSSION

Many studies have demonstrated that meteorological factors and ambient pollutants were associated with influenza seasonality, but results were inconsistent among studies especially for those conducted in regions of different climatic zones.^16^, ^18^, ^22^, ^23^, ^31^, ^32^ Here, we included a total of 20 165 laboratory‐confirmed influenza cases in our analysis and used statistical models to explore potential environmental drivers of seasonal oscillations of influenza in a city located in a subtropical area, Hangzhou, Zhejiang province, China. In brief, we found high temperature, wet and dry conditions, and heavy rainfall are the major risk factors associated with a higher risk of influenza infections from 2014 to 2018. These findings shall be essential for the step‐up of influenza surveillance and early warning systems to facilitate vaccination campaigns.

Compared with RH, AH is regarded as a humidity measure independent of the ambient temperature. Previous studies have shown the prediction power of AH was stronger than that of RH.^7^, ^8^ Shaman and Kohn^7^ showed that low AH would increase the transmission efficiency of the influenza virus in both indoor and outdoor environments through facilitating its survival, but the underlying mechanism remained poorly understood. Another epidemiological study conducted in a temperate climate region in the United States found supporting evidence that low AH in the prior weeks was associated with increased wintertime influenza‐related mortality.^8^ In spite of the aforementioned, high rather than low AH was shown to be associated with increased influenza activity in a tropical setting.^33^ As our study showed both low and high AH were associated with increased influenza activity, we speculate that influenza activity in Hangzhou might be influenced by the subtropical weather of the region. Surprisingly, the pattern of the relationship is similar to that of other infectious diseases (such as COVID‐19) and humidity metrics.^34^

In this study, we found a statistically significantly higher influenza activity when the mean weekly RH was low. Our result echoed the guinea pig study by Lowen et al^5^, which showed low RH favors influenza transmission. An experimental study by Yang et al^35^ claimed that the viability of the influenza A virus in all media and mucus was highest when RH was below ∼50%. This finding might be explained by the high viability of the influenza A virus in droplets at low RH and an enhancement of viral survival in dry conditions.^36^ However, we noticed two studies which reported contradictory findings regarding the effect of high RH. Lowen et al^5^ reported complete blockage of transmission at RH > 80% but Yang et al^35^ reported very high viability of influenza viruses at RH close to 100%. Our results which showed insignificant risk of influenza infection at high RH seem to agree more with the virus transmission mechanism proposed by Lowen et al.^5^ This underscores the need for further studies to examine the effect of RH on disease transmission at population level.

Similar to the findings in subtropical cities such as Hong Kong and Okinawa,^9^, ^29^ we found a positive association between high temperature and influenza activity although previous studies reported that cold ambient temperature was associated with influenza transmission in temperate regions.^4^, ^5^, ^12^, ^18^, ^37^ Lowen et al^5^ indicated that low temperature could increase the duration of virus shedding and thus enhance influenza transmission. From a global perspective, an epidemiological study indicated that monthly influenza positivity percentage was associated with low monthly mean temperature after adjusting for sunshine, precipitation, AH, and latitude.^37^ Similar results were also reported in other subtropical settings such as Brisbane and Hong Kong.^38^, ^39^ In this study, we showed that in Hangzhou, higher influenza activity was associated with lower weekly mean temperature, although the association identified did not reach statistical significance. As suggested by Lowen and Steel,^40^ we speculate the inconsistency regarding the impact of RH in subtropical settings was due to the mediation effect of temperature given virus transmission efficiency varies largely even under moderate temperature conditions. Another global study also indicated that the nature of association identified could vary across cities even if they are in the same climate zone.^36^ Apart from that, the lagged effect of high temperature is evident in a study conducted on the children population in another subtropical setting in China.^41^ Since cold conditions in subtropical settings might only have limited effect on hosts’ activities (e.g. using indoor heating),^36^ the role of temperature in influenza seasonality might be more complicated in the subtropics than in temperate settings.

Rainfall exhibited a positive association with influenza activity in our study, which is also observed in studies conducted in tropical regions such as Singapore,^13^ and subtropical regions such as Hong Kong.^12^ According to Soebiyanto et al,^12^ the role rainfall played in influenza transmission is neither on altering virus survivorship nor host susceptibility. Rainfall promotes changes in social behavior of hosts, who might prefer indoor activities on rainy days, hence favors contact transmission.^12^

Some studies suggested a significant positive association between PM_2.5_ and influenza activity,^19^, ^21^, ^22^ hypothesizing that the influenza virus can travel farther by attaching to fine inhalable particles which help deliver the viral agents to the respiratory epithelial cells.^42^, ^43^ Although the risk of influenza increased along with increasing PM_2.5_ concentration in our study, the association was marginally statistically insignificant. One possible reason might be over‐smoothing due to the use of weekly rather than daily data. According to the epidemiological study conducted by Huang et al^23^ which explored the association between PM_2.5_ and influenza in another subtropical city, the effect of PM_2.5_ exposure on daily influenza‐like cases peaked at lag 0 and remained to be statistically significant only up to a lag of 3 days. Thus, the one‐week lag period defined in our study might be slightly too long which diluted the effects of PM_2.5_. It could also be that the four‐year study period was not long enough to render sufficient statistical power to detect the relationship between the two.

This study has several limitations. First, although the CISDCP is the world's largest web‐based notifiable infectious disease reporting system which collects real‐time information on disease incidence,^24^ underreporting of influenza cases might still occur because not all infected patients, for example patients with mild influenza symptoms, would visit the surveillance hospitals.^17^ Second, although school holidays were included in our model to adjust for human encounters and social mixing patterns,^26^ other confounders might still exist. For example, this study did not take influenza vaccination into consideration and this might cause confounding bias. Despite the fact that annual influenza vaccination coverage was only 2%‐3% in China,^44^ vaccination remains to be the most appropriate way to prevent influenza, hence plays an important role in annual influenza epidemics. Third, we did not conduct subgroup analysis by influenza types due to a lack of type‐specific data. Previous studies have shown that the impacts of meteorological variables on influenza activity could vary by types.^10^, ^45^ Fourth, this study is a retrospective time series analysis and further studies should be conducted to validate our results. For example, plausible causality and potential interactions, such as effect modification between PM_2.5_ and temperature, should be further investigated.^22^ All in all, this study took exposure‐lag‐response association into account and explored the potential environmental drivers of seasonal oscillations of influenza in a subtropical area.

## 5 CONCLUSION

Our study showed that high temperature, wet and dry conditions, and heavy rainfall were the major risk factors associated with higher risk of influenza infections. In line with other subtropical settings, the effects of meteorological variables on influenza activity documented in existing literature are not as consistent as that in temperate settings; this study thus offers additional understanding on their effects on influenza activities. Our findings shall be useful for the development of influenza surveillance and early warning systems.

## 6 ETHICAL APPROVAL AND CONSENT TO PARTICIPATE

This study was reviewed and approved by the Medical Ethics Committee of the Zhejiang Centre for Disease Control and Prevention (CDC). Informed consents were exempt from the ethics committee in accordance with the CDC policy of continuing public health investigations of notifiable infectious diseases.

## CONFLICT OF INTEREST

The authors declare they have no actual or potential competing financial interests.

## AUTHORS’ CONTRIBUTIONS

XL, WC, ZY, and EC: Data collection; SYFL and KCC: Data analysis; XL, SYFL, and KNM: Manuscript drafting and writing the final version; MW, BCYZ, and KCC: Manuscript revision; All authors: Manuscript reading and approval.

## AUTHOR CONTRIBUTION

**Steven Yuk‐Fai LAU:** Formal analysis (equal); Investigation (equal); Methodology (equal); Software (equal); Validation (equal); Visualization (equal); Writing‐review & editing (equal). **Wei Cheng:** Data curation (equal); Resources (equal); Writing‐review & editing (equal). **Zhao Yu:** Data curation (equal); Resources (equal); Writing‐review & editing (equal). **Kirran MOHAMMAD:** Investigation (equal); Validation (equal); Writing‐review & editing (equal). **Maggie H Wang:** Conceptualization (equal); Funding acquisition (equal); Project administration (equal); Writing‐review & editing (equal). **Benny Chung‐Ying ZEE:** Conceptualization (equal); Project administration (equal); Supervision (equal); Writing‐review & editing (equal). **Xi LI:** Data curation (equal); Formal analysis (lead); Methodology (equal); Writing‐original draft (equal). **Ka Chun Chong:** Conceptualization (equal); Investigation (equal); Methodology (equal); Project administration (lead). **Enfu Chen:** Conceptualization (equal); Data curation (equal); Project administration (equal); Resources (equal); Supervision (equal); Writing‐review & editing (equal).

## Funding information

The work is supported by National Natural Science Foundation of China (71974165 and 81473035).

### PEER REVIEW

The peer review history for this article is available at https://publons.com/publon/10.1111/irv.12829.

## Supporting information

FigS1

## Data Availability

The datasets are available from the corresponding author on reasonable request.
