# Supplementary material for: Independent association between meteorological factors, PM2.5, and seasonal influenza activity in Hangzhou, Zhejiang province, China
Source: Influenza Other Respir Viruses. 2020 Dec 20;15(4):513–20. doi: 10.1111/irv.12829 (PMC8189232; doi:10.1111/irv.12829)

**Figure S1.** Adjusted relative risk (ARR) and corresponding 95% confidence interval for influenza activity at different levels of pollution and meteorological conditions when the lags were set to 2 weeks.
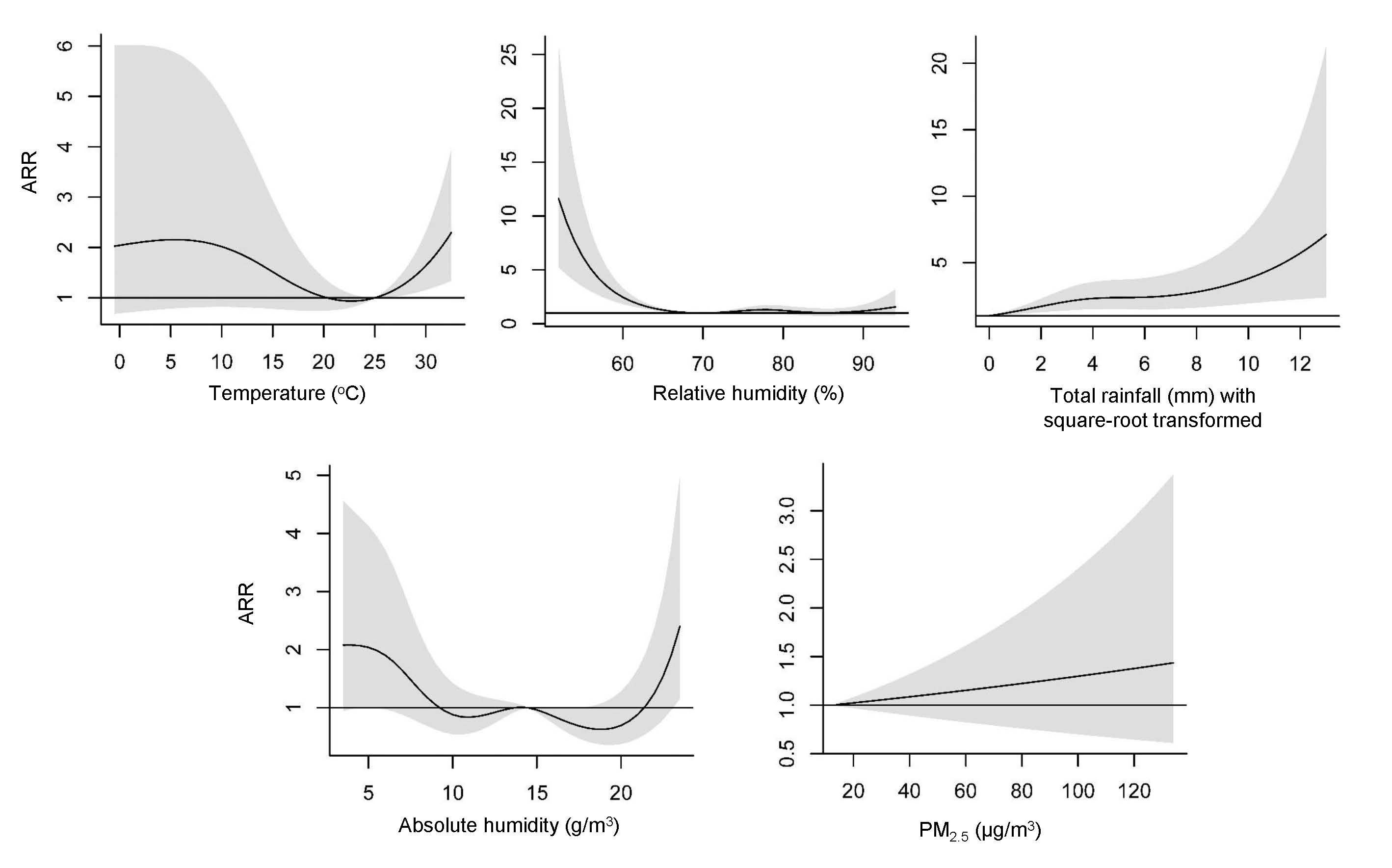

Supplement: Supplementary file 1 — FigS1 [file IRV-15-513-s001.docx]
